# How do emergency departments respond to ambulance pre-alert calls? A qualitative exploration of the management of pre-alerts in UK emergency departments

**DOI:** 10.1136/emermed-2023-213854

**Published:** 2024-09-17

**Authors:** Jaqui Long, Fiona C Sampson, Joanne Coster, Rachel O’Hara, Fiona Bell, Steve Goodacre

**Affiliations:** 1 SCHARR, School of Medicine and Population Health, The University of Sheffield, Sheffield, UK; 2 Yorkshire Ambulance Service NHS Trust, Wakefield, UK

**Keywords:** emergency departments, pre-hospital, qualitative research, health service accessibility

## Abstract

**Background:**

Calls to emergency departments (EDs) from ambulances to alert them to a critical case being transported to that facility that requires a special response (‘pre-alerts’) have been shown to improve outcomes for patients requiring immediate time-critical treatment (eg, stroke). However, little is known about their usefulness for other patients and the processes involved in ED responses to them. This study aimed to understand how pre-alerts influence patient care in the ED.

**Methods:**

We undertook non-participant observation (162 hours, 143 pre-alerts) and semi-structured interviews with staff (n=40) in six UK EDs between August 2022 and April 2023 focusing on how ED staff respond to pre-alert calls and what influences their response. Observation notes and interview transcripts were imported into NVivo and analysed using a thematic approach.

**Results:**

Pre-alert calls involved significant time and resources for ED staff but they were valued as they enabled staff to prepare for a patient’s arrival (practically and psychologically). High demand and handover delays at ED created additional pre-alerts due to ambulance clinician concerns about the impact of long waits on patients.

Despite the risk of pre-alert fatigue from calls for patients considered not to require a special response, ED clinicians appreciated timely pre-alert information, perceiving a higher risk from underalerting than overalerting. Variation in ED response was influenced by individual and organisational factors, particularly the resources available at the time of pre-alert. Unclear ED processes for receiving, documenting and sharing information about pre-alerts increased the risk of information loss.

**Conclusion:**

Improving processes for receiving and sharing pre-alert information may help ED clinicians prepare appropriately for incoming patients. Alternative routes for ambulance clinicians to seek advice on borderline pre-alert patients may help to improve the appropriateness of pre-alerts.

**WHAT IS ALREADY KNOWN ON THIS TOPIC**

- Ambulance pre-alerts can help emergency department (ED) staff to prepare for a patient’s arrival and can lead to improved outcomes for patients requiring immediate senior review on arrival.
- Research about pre-alert practice focuses on outcomes for patients who have been pre-alerted but there is a lack of evidence about the effect of pre-alerts on ED staff and patient care to identify potential good practice and areas for improvement.

**WHAT THIS STUDY ADDS**

- Pre-alerts placed additional demands on ED resources but were valued in terms of enabling both practical and psychological preparedness.
- Variation in ED processes, layout and capacity led to different ED responses to pre-alert calls, particularly for patients who were not brought into resus.

**HOW THIS STUDY MIGHT AFFECT RESEARCH, PRACTICE OR POLICY**

- Standardisation of processes for improving flow and assessing high-risk patients may help reduce variation in the ED management of pre-alerted patients.
- Improving awareness of the complexity of ED pre-alert decision-making may help improve ambulance clinician pre-alert practice.

## Introduction

 Ambulance clinicians may use a pre-arrival call to the receiving hospital (pre-alert) when they consider a patient requires a special response.^1^ Evidence suggests that pre-alerts lead to improved patient outcomes for certain conditions where patient pathways indicate the need for a specific and timely response, for example, initiation of treatment, preparation of trauma team personnel.^2^,^8^ Guidelines for the management of patients with major trauma, sepsis, stroke and cardiac arrest all include recommendations for pre-alert use.^9^,^13^ More recently, UK guidance published jointly by the Royal College of Emergency Medicine and Association of Ambulance Chief Executives specifies other conditions or physiological criteria that should be considered for pre-alert.^1^

In the current context of high emergency department (ED) crowding and long ambulance handover times, pre-alerts can potentially ensure that critically ill patients bypass ambulance queues and receive timely care.^1 14^ However, there are concerns that unnecessary pre-alerts can divert resources away from where they are most needed, slow patient flow and contribute to pre-alert fatigue.^15^,^18^

Despite recognition of the need for pre-alert practice to maximise the patient benefit but minimise demands on limited ED resources,^10 12^ there is a lack of research examining how pre-alert practice influences patient care in the ED and the impact on ED staff and patients. As part of a mixed methods study exploring the impact of pre-alerts on ED and ambulance staff and patients, we undertook qualitative research to explore how pre-alerts influence patient care in the ED.

## Methods

We used a qualitative design incorporating semi-structured interviews and non-participant observation.

### Context and sampling strategy

Six ED sites in the UK were identified by selecting one major trauma centre (MTC) and one trauma unit (TU) within each of the three ambulance services, focusing on those with high numbers of pre-alerts to ensure that sufficient pre-alert activity could be observed. Sites were selected to cover diverse populations in terms of deprivation, rural/urban mix and diverse ethnic populations.

Non-participant observations and informal conversations with ED staff were undertaken at each site. We recruited ED staff for interviews primarily through direct invitation during observations, with local investigators also asked to invite staff with particular roles (eg, clinical director). We aimed to recruit a range of different roles at each site including senior and junior medical and nursing staff as well as other roles identified as important by individuals.

### Data collection methods

Observations and interviews were undertaken principally by two researchers (JL and JC) with some dual observations where departments were particularly large or busy to observe multiple areas. Researchers were principally based near to the pre-alert phone (usually in resus) in order to be able to observe and record the ED response to calls, but also observed throughout the ED and the ambulance waiting areas. Staff were made aware of the presence of researchers and given the option to opt out of being observed. Further details of observations are available in online supplemental table 1.

### Data collection instruments, technologies and processing

We developed an interview topic guide in collaboration with our project management and patient and public involvement (PPI) groups (see online supplemental material 2). Topic guides were followed flexibly. Observation guides were developed and refined after initial visits including a form to record key details of individual pre-alert calls (excluding any patient data). Interviews were conducted online or by phone and recorded using encrypted dictaphones and transcribed verbatim. Data was stored in a secure restricted access university filestore, accessible only by the research team at the University of Sheffield. All participants were allocated a unique code which was used within data excerpts. Transcripts were not made openly available to protect anonymity. All fieldwork data (interview transcripts and observation notes) were imported into NVivo (qualitative data analysis software).^19^

### Researcher characteristics and reflexivity

The researchers involved in the data collection were experienced researchers working in health services research with social science/psychology background but not clinically trained. Two of the researchers (FCS and RO’H) had prior experience of undertaking non-participant observation in emergency settings while the researchers involved in fieldwork (JL/JC) had no prior experience and thus fewer preconceptions about the research topic. Observation notes were written up in detail shortly after the observation took place to incorporate researcher reflections and interpretation of events.

### Data analysis

Data was analysed using a thematic approach according to the principles of Braun and Clark.^20^ Data familiarisation involved RO’H, JL, JC and FCS reading a subset of the interviews and developing initial themes and an initial coding framework. Data was coded independently, undertaken initially by RO’H (who had not undertaken any fieldwork) and JL (who had done the majority of data collection). Coding was discussed and refined within the wider group on a weekly basis in order to refine the analysis. Codes and changes to coding frameworks were documented at each stage. Code summaries were developed and cross-cutting themes were identified after discussion between the group.

### Techniques to enhance trustworthiness

Researchers clarified points and summarised findings during interviews to ensure a shared understanding of the data. Researcher triangulation within both the data collection and analysis phases helped improve the trustworthiness of the analysis. Results were presented to the PPI group at an online workshop and their views of the findings and which findings they considered most important contributed to a wider stakeholder workshop incorporating research participants and key stakeholders from ambulance service and ED national bodies on how to use the findings to improve practice.

### Patient and public involvement

We created a study-specific PPI group which included people who had experience of being pre-alerted to the hospital and of accessing ambulance and ED care. The group met regularly throughout the study. The PPI group reviewed and discussed the interview schedules and the emerging findings from the interviews. The group’s experience of pre-alerts also helped to inform observation practice.

## Results

We undertook interviews with 40 ED clinicians including doctors, nurses and advanced clinical practitioner roles over six sites (three MTCs and three TUs) (table 1).

**Table 1:** Characteristics of ED clinician interview participants

ED	Major trauma centre	Site A	7
		Site D	8
		Site E	6
	Trauma unit	Site B	4
		Site C	10
		Site F	5
Role		Consultant	16
		Registrar	7
		Junior doctor	2
		Senior nurse	10
		Nurse	2
		Advanced practitioner*	3
Years in role		<1 year	8
		1–5 years	20
		6–10 years	6
		>10 years	6
Gender		Female	23
		Male	17
Ethnicity		White British	33
		British Asian	4
		Not reported	3

* Advanced practitioners include nurses and paramedics with advanced training to work in ED and resus/trauma settings.

EDemergency department

We undertook 25 sessions of observations across six EDs completing a total of 162 hours (or 123 hours of actual observed time) and observing 143 pre-alert calls (see online supplemental table 1). Sessions ranged from 2.25 to 7.25 hours, average 5 hours. Descriptions of each site and their processes for managing pre-alerts are available in onlinesupplemental tables 3  4. We identified five key aspects of EDs practice in response to pre-alert calls:

- Pre-alerts were valued in enabling preparedness but used significant ED resources.
- ED crowding and ambulance queues contributed to an increased perceived need to pre-alert.
- The challenge of balancing the risks associated with underalerting and overalerting.
- Variation in pre-alert response was influenced by individual and organisational factors within ED settings.
- Lack of clearly defined processes for receiving, documenting and communicating pre-alerts affects the usefulness of pre-alerts.

### Pre-alerts were valued in enabling preparedness but used significant ED resources

Pre-alerts enable staff to prepare physically and psychologically for the pre-alerted patient’s arrival, judging patient needs based on the information communicated during the call. In the context of high ED demand, creating space for incoming patients often involved relocating critically ill patients who would otherwise have remained in resus, while also trying to protect space for potentially more critical cases. This ‘juggling available space and staff’ (ED56, consultant) was repeatedly observed and commented on.

Having that information beforehand is really handy because if our resus room is full then we need to kind of step people down into the majors area or […] maybe move them further down the resus rooms so we’ve got an airway bay free. (ED03, senior nurse)

Pre-alert calls involved significant staff resources and potential risk for other patients when staff or other patients were moved in response to pre-alert calls.

A pre-alert is not a harm free intervention. Sometimes in resus there is only 1 spare bed. And a pre-alert might take a reg[istrar], ED nurse, anaesthetist, consultant, [allied health professional]. (Site D MTC Obs 3a)

Responding to the pre-alert phone was always observed to be prioritised by staff regardless of the level of demand at the time of the call. The call also influenced the behaviour of other nearby staff who often listened in, read what was being written and sometimes started to act before the call was complete. Resource demands were greater for complex cases (eg, major trauma) or where immediate life support was requested involving the readying of equipment and calling specialist teams from elsewhere in the hospital. For example, the following pre-alert case involved 17 staff over a half-hour period.

10:57 - Consultant passing info [about pre-alert] to nurse and doctor waiting by pre-alert phone—patient is on their way. Conversations between various members of staff. Equipment and trolleys moved so there is room for people. Ultrasound wheeled over.

11:04 - Consultant pushing screens back for more space. Staff put on their role labels. More info is added to the board. Call going out to the trauma team and cardiothoracic. Sister checking notification has gone.

11:08 - People start arriving—major trauma consultant, radiographer, others, discussing plan of action. Five staff in the bay, another five by reception.

11:14 - Formal briefing. Now 17 staff in the area waiting. Going through basics, what needs doing, by who. Consultant asks if there are any questions. Checking the neurosurgeon, agree to call once the patient is here. People discussing roles. Consultant is discussing treatment plan. People asked to sign in on the checklist.

11:24 - Patient arrives.

Extract from observation notes (site A MTC Obs 1)—impact of pre-alert on staff resources.

Due to the workload involved in acting on pre-alerts, timing of the call was perceived as critical to maximising the benefit for both patients and staff, particularly when substantial preparation was needed. ED clinicians expressed frustration at underestimated arrival times resulting in wasted resources especially when large teams of specialist staff had been assembled in anticipation of the imminent arrival of a time-critical patient. Short notice calls (less than 5 min) were also challenging, but ED staff valued some advance warning despite expressing frustration at very last-minute pre-alerts and where ambulance clinicians did not pre-alert because they were nearby.

I would rather a crew rung me and said, I’m two min away. I’ve got a really poorly one, can you look at them? So then at least I’ve got those two min to mentally say like, right, this patient can move here, this patient can move here. (ED49, senior nurse)

12:50 Critical care paramedics bring a patient in who wasn’t pre-alerted. Apparently this happens often. I asked them why they didn’t pre-alert and they said it was because they were only 2 minutes away. (Site E MTC Obs2b)

Even when staff did not make any immediate practical change within the department in response to the pre-alert, they still valued the pre-alert as enabling them to mentally plan and prepare. Even short-notice pre-alerts enabled some mental preparedness through awareness of the risk profile of patients arriving into the ED.

Part of the whole essence of pre-alerts is for your own mental model, isn’t it? It’s for your own preparation. (ED18, registrar)

You are aware of what’s there, it’s not a hidden risk, it’s a, you’re aware of the risks. (ED43, consultant)

### ED crowding and increased ambulance wait times contributed to an increased perceived need to pre-alert

During fieldwork, we found that EDs often had high levels of crowding and ambulance queues. Pre-alerts were perceived by ED staff to be more important in this context due to the increased risk for patients waiting on ambulances, and were considered necessary in anticipation of likely deterioration. However, ED crowding and patient queues were also identified as contributing to an increase in unnecessary pre-alert calls where ambulance clinicians had concerns about the patient having to wait in the ambulance and/or wanting advice. This was thought to result in a higher volume of calls to the pre-alert phone, increasing workload and pressures for EDs.

I think the pre-alert is even more important now, because it makes the difference between knowing you’ve got to find a space that is gonna be hard to find for someone in an immediate time frame, vs them being able to wait for twelve hours on the back of a truck. (ED18, registrar)

Now, we get a phone call where actually it’s not really a critically ill patient. It’s just for advice, you know “What do we do with…?” and this has come out of the fact that, if they can’t drop the patient off in resus, then we are committing them to queueing up in the corridor for hours. (ED46, consultant)

While acknowledging the validity of ambulance clinician concerns and potential patient safety benefits, individual clinician attitudes towards these calls and perceptions of what the ‘red phone’ should be used for caused some frustration and tensions due to the increase in workload created. Some commented that advice calls should be managed elsewhere, potentially via an alternative line or ambulance service support.

I know we get quite a few where they may be more junior paramedics who will ring up for advice on the pre-alert phone to say y’know I’m not sure if this needs pre-alerting, but this is what I’ve got. Which of course, nobody minds that, it’s just difficult. If you’ve got a busy resus, and that phone’s constantly going, and they’re not pre-alerts. So it’s almost like we need a pre-alert phone and an advice phone. (ED55, senior nurse)

When pre-alerted patients were sent to the usual ED entrance, they were often prioritised by offering an immediate quick review to ensure that nothing significant had been missed during the pre-alert call. One site also offered this for patients who had not been pre-alerted, meaning that ambulance clinicians could receive immediate reassurance about a patient they had concerns about without the need to pre-alert.

Pregnant lady in resus not pre-alerted by double technician crew because ‘if it’s borderline we normally ring the doorbell and see what they want’. ‘Turn up and check’ has been observed at least three times now, maybe as the ambulance door is close to resus so it’s easy to stop and check as the crew are walking through to [usual ED area]. (Site E MTC Obs 3b)

### The challenge of balancing the risks associated with underalerting and overalerting

ED clinicians favoured a risk-averse approach to pre-alerting, whereby ‘it’s better knowing about them than not knowing about them’ (ED4, registrar). They also identified specific conditions as being underalerted, for example, elderly (silver) trauma. Being given information about borderline cases enabled them to prepare for potential deterioration and the chance to ‘eyeball’ a patient if necessary but did not mean they were required to act on the information.

If you’re being pragmatic about it, any information is useful. And I think that just because they’re pre-alerted doesn’t necessarily mean we have to respond in a way that they would perceive they would want to be responded to. (ED30)

Pre-alert information and judgements based on telephone communication could involve a degree of ambiguity but the consequences of underalerting were felt to be more serious than those of overalerting, providing no opportunity to identify and prepare for a time-critical patient.

In any triage system you have to overtriage, if every call you’re bringing in is entirely appropriate then you’re missing things. Anything that encourages people to alert less, it’s fraught with danger. (ED38, registrar)

However, ED staff raised concerns about the risk of pre-alert fatigue where pre-alert calls may not be taken as seriously if overused. This was raised particularly in relation to sepsis where ambulance clinician protocols were regarded as more risk-averse and recommended pre-alerting but ED staff were confident that the patient could be managed outside of resus. Within fieldwork, we observed a number of pre-alerts where pre-alert calls for suspected sepsis were sent elsewhere in the ED rather than resus.

The classic red flag sepsis is the one that gets our eyes rolling most of the time, to be honest. Most patients with sort of sepsis probably don’t need to be in resus. (ED5, consultant)

[Practitioner] says ‘there’s a lot of pre-alert fatigue here. A lot of sepsis that really aren’t that unwell. When they arrive their numbers are okay and they’re sat up texting. And you want to say, what needs resuscitating here? We get sepsis after sepsis after sepsis. We get annoyed and then they get annoyed at us because we’re not letting patients in.’ (Site D MTC Obs 3a)

### Variation in pre-alert response was influenced by individual and organisational factors within ED settings

Potential pathways for pre-alerted patients varied across the sites reflecting different approaches to managing risk, historical processes and capacity, layout and resourcing of the ED (see online supplemental table 3). Over one-third of the pre-alerts observed (53/143) were not sent to resus or similar high-level care. Pre-alert responses varied between different ED clinicians depending on their experience, attitudes to risk and situational awareness. Trust in the information provided during pre-alert calls was potentially influenced by established relationships with ambulance clinicians.

Certainly if the senior paramedic’s brought the patient in and they’re saying they’re really poorly I’ll listen to them. I mean that’s just, if they’re concerned about the patient then I’m concerned as well. (ED42, consultant)

Conversation with [role]: Some crews alert everything, whereas others if they say someone is sick, you know they are sick, take it seriously whatever the obs. (Site A MTC Obs 5a)

ED staff commented that while ambulance clinicians may have expectations regarding the ED response to their pre-alert (eg, placed in resus) there are a number of factors that may mean their expectations are not met. The ED resources available at the time of call influenced the ED response (ie, where they told the ambulance clinician to bring the patient). When EDs were busy, this involved balancing patients that were ‘least sick’ (rather than ‘most well’) and/or directing all but the most critically ill patients to a different area of the ED. During the majority of observations, pre-alert demand was high with repeated calls in quick succession creating notable pressure on staff and space, particularly when space in other areas of the ED and wider hospital was limited.

Clinician in charge arrives—“I hate shifts like this, who’s coming out?” and says “they’re trying to work out who’s the least worst, not who’s well enough to move out, but who’s the least likely to arrest on me if I move them out there.” (Site D MTC Obs 4a)

Conversation about the two pre-alerts [that had just been called in]. ‘What’s yours? Is yours more important than mine? We’ve only got one space.’ (Site D MTC Obs 1a)

### Lack of clearly defined processes for receiving, documenting and communicating pre-alerts affects usefulness of pre-alerts

Experience and seniority were regarded as important in understanding what information from the ambulance clinician would influence where the patient should be seen on arrival. Despite an understanding that it was preferable for more senior clinicians (medical or nursing) to answer pre-alert calls, preferably also one with an oversight role in the department aware of resource availability, many EDs lacked formal policies for who should answer a pre-alert. During observations, responses from less experienced staff required input from a senior clinician, sometimes in the background, directing the call taker on what to ask or to advise.

F2 [doctor] answers, Nurse in charge comes and stands behind. Consultant and more senior reg also there—unintentional overdose of codeine. The consultant circles GCS [Glasgow Coma Scale] on the form to indicate what to ask. Consultant says he can go to [usual ED entrance]. (Site F TU Obs 2)

If it’s really busy, junior doctor or junior sister/staff nurse will answer but they sometimes will forget to ask something even though it’s on the sheet so I think that’s why it is quite important that it is a senior member of the team or, say if I’m present, at least I’m stood next to them to kind of prompt them ’ (ED3, senior nurse)

Processes for documenting, managing and sharing pre-alert information varied. Observations and interviews identified the potential for this to result in clinicians being unaware of pre-alerted patients and the inability to identify from case notes whether a patient was pre-alerted (see online supplemental table 4). Pre-alert information was communicated to staff within the ED verbally (bleep or face-to-face) or via written information taken to the receiving area but the information was not always consistently conveyed, particularly when patients were not brought into resus. Where processes for information flow following the pre-alert were less clear or where information flow was interrupted by other pressures, this could lead to pre-alerts not being communicated effectively, causing frustration for both ED and ambulance clinicians.

Some of our junior doctors, they’ll just take the call, they won’t tell the crew where to go. And sometimes what they haven’t done, or done in the past, is just left the form on my desk and not told me about it, and we’ve had crews rock up with an alert and I don’t know about them. (ED33, senior nurse)

Communication could be much better to make sure that everybody knows that an alert is coming in and because sometimes alerts arrive and nobody but the nurse in charge and the nurse in the area is expecting them. There are times when I don’t know anything about an alert and it will arrive in the department and or you’ll find that there’s an alert on an ambulance that we didn’t know about. (ED36, consultant)

## Discussion

We identified a complex range of factors influencing pre-alert practice within EDs. Pre-alerts were highly valued by ED staff as enabling them to plan both practically and psychologically for a patient’s arrival and to manage wider patient flow within the department. They also involved significant work for ED staff prior to the arrival of the patient which amplified the importance of accurate estimated arrival times and high-quality pre-alert information. Staff reflected on the challenge of balancing the benefits of pre-alerts with the impact on ED resources including the risks associated with both overalerting and underalerting. We observed variation both within and between EDs in the processes and practices at each stage of managing the pre-alert response. Organisational and individual sources of variation included resource availability at the time of the call, the availability of alternative options for managing pre-alerted patients not sent to resus, the experience of ED staff involved and individual ED processes for managing pre-alert information.

Existing pre-alert literature focuses on assessing improved outcomes for specific patient groups who are pre-alerted.^2^,^721^ Our findings identified that EDs respond to a wide range of pre-alerts by preparing space, personnel and equipment but also preparing psychologically for the patient’s arrival which is key to ensuring safe patient flow. We identified that pre-alerts did not always result in a resus bed, particularly when resources were pressured, but that EDs frequently responded in other ways which increased their priority and helped staff to manage the associated excess risk. These processes included ‘eyeballing’ patients on arrival or putting them in a higher care area. Sujan *et al* reported that pre-alerts had an important anticipatory function in enabling EDs to prepare for the patient’s arrival.^18^

ED crowding and prolonged waiting times are associated with increased mortality and a negative impact on other patient outcomes.^22^ We found EDs were frequently crowded, operating beyond capacity with long ambulance queues and ED staff were often unable to create space in resus for ambulance patients who would otherwise have been considered to warrant a resus bed. While these patients were still usually considered higher risk within the ED clinician’s mental model of patients within the department, this may mean that ambulance clinicians perceive their pre-alerts not to have been responded to adequately. Coster *et al* reported that a third of ambulance clinicians surveyed reported that enabling the ED to make a space in resus was always a factor in making pre-alert decisions.^23^ This is particularly problematic given that ambulance clinician understanding of pre-alert decision-making is influenced by the ED response to previous pre-alerts^24^ and may lead to ambulance clinicians questioning the appropriateness of their decisions or feeling undermined and their decisions not respected.^24 25^ Although we did identify concerns about ‘pre-alert fatigue’ due to perceived overalerting, this did not largely appear to affect ED clinician’s behaviour and pre-alerts were generally taken seriously and prioritised. Overalerting for sepsis may reflect the poor predictive value of diagnostic impression and early warning scores for sepsis and limitations of the sepsis diagnostic definition in a typical ED population.^26^ Overalerting did not appear to generate significant risks to other patients because ED clinicians made their own assessment of the patient’s needs and provided a graduated response, only freeing up space in resus when it was safe and necessary to do so. Berglund *et al* identified that stroke pre-notification improved time to thrombolysis with no negative impact on other pre-hospital patients.^27^ Brown and Bleetman identified underalerting to be a greater problem than overalerting in a small sample of 52 critically ill patients of whom 29 were not alerted.^28^ They presented an ideal model of pre-alerting that included all critically ill patients plus some non-critical patients to allow for a ‘margin of error’. Sheppard *et al* identified a lower proportion of patients not pre-alerted for stroke who should have been compared with those pre-alerted who should not have been.^17^

Pre-alerts were shown to take up staff time and generate interruptions to caring for patients already in the ED. Unnecessary interruptions and inappropriate pre-alerts were a source of frustration for ED staff. Despite this, pre-alert calls were usually perceived to be beneficial. Interruptions are common in the ED and can impact negatively on patient safety. However, interruptions may also be beneficial when providing critical time-sensitive information relating directly to patient care as occurs with pre-alerts.^29 30^

### Limitations

Although we aimed to represent a diverse population, there may be limitations to the transferability of findings. The three ambulance services primarily followed a ‘direct to ED’ call model whereas some other ambulance services use a call desk model. Half of our sample were designated MTC which were perceived by ambulance clinicians in our fieldwork to manage pre-alerts better than local EDs where pre-alerts are less frequent. Observations were undertaken at times that ambulance data identified that most pre-alerts occurred. This meant we undertook few observations at night or on weekends when staffing levels and case mix may differ. It is also possible that some behaviour modification took place as a result of our presence as observers. However, due to the busyness of the EDs observed and the high number of staff often surrounding the call-taker, we do not feel that the impact of our presence was significant. Other studies suggest that while the presence of researchers could initially make staff more mindful of their practice, they would quickly resume focus on normal patient care activities.^31 32^ Although we reached saturation during the analysis of observations and interviews for the main themes, we lacked data to explore certain themes (eg, role and seniority) further.

### Implications of the results for practice or policy

While a considerable level of variation in response to pre-alert calls is outside the control of ED staff, particularly in the current context of high demand, there is potential to increase consistency in some areas. Unwarranted variation in healthcare is a key challenge to healthcare policymakers.^33^ Some of the identified sources of variation are necessary but some may negatively affect the usefulness of pre-alerts and could be addressed by implementing more clearly defined processes for receiving, documenting and communicating pre-alerts and ensuring these are disseminated to all relevant staff.

Simple guidance and training could help EDs review and clarify their practice in relation to who answers pre-alert calls and how; who makes decisions and how; what information is documented; how information regarding the pre-alert is communicated to others including when they are not accepted into resus. There is also a need to ensure decisions and protocols are disseminated to all staff, potentially through brief training or other mechanisms. This is particularly important given the rapid turnover and frequent rotation of staff within EDs.

There is a need for further alignment of ED and ambulance service policies and pre-alert thresholds for some conditions and for identifying routes for ambulance clinicians to seek advice on patients they are uncertain about. There is also a need to increase ambulance service awareness of the complexity of ED decision-making regarding pre-alerts to avoid misunderstanding and tension when ambulance clinicians do not receive an anticipated or consistent response which could impact negatively on future pre-alert behaviour. Increasing consistency in receiving and sharing pre-alert information may help ED clinicians prepare appropriately for an incoming patient.

## supplementary material

10.1136/emermed-2023-213854 online supplemental file 1

10.1136/emermed-2023-213854 online supplemental file 2

10.1136/emermed-2023-213854 online supplemental file 3

10.1136/emermed-2023-213854 online supplemental file 4

## Data Availability

No data are available.
