# Supplementary material for: How do emergency departments respond to ambulance pre-alert calls? A qualitative exploration of the management of pre-alerts in UK emergency departments
Source: Emerg Med J. 2024 Sep 17;42(1):e213854. doi: 10.1136/emermed-2023-213854 (PMC11874362; doi:10.1136/emermed-2023-213854)
Supplement: online supplemental file 1 [file emermed-42-1-s001.pdf]

Supplementary table 1: Details of pre-alerts observed

Site	No. alerts observed	Type of alert	No. hours observed (over no. sessions)	Seen in resus/ high care*	Directed to usual ED entrance++	Senior clinician triage	No other
A* (MTC)	26	21 medical, 5 trauma	31.5, (5)	15	11	0	0
B (TU)	6	6 medical, 0 trauma	14, (3)	5	0	1	0
C (TU)	34	27 medical, 7 trauma	28, (6)	16	0	11	7
D* (MTC)	28	26 medical**, 2 trauma	35.5, (4)	20	0	5	3
E* (MTC)	24	19 medical, 5 trauma	25, (3)	18	0	1	5
F* (TU)	25	25 medical, 0 trauma	28, (4)	16	8	0	1

* includes some hours double observation (A=8hrs; D=18hrs; E=8hrs; F=5hrs)

** one alert classed as both medical and trauma

+High care refers to areas of the ED that were more highly staffed than the 'majors' department but less well resourced than resus.

++ The term 'usual ED entrance' is used to describe all departments' main ambulance entrance and initial assessment and treatment area, i.e. where patients not being taken to resus etc are received.
