# Supplementary material for: How do emergency departments respond to ambulance pre-alert calls? A qualitative exploration of the management of pre-alerts in UK emergency departments
Source: Emerg Med J. 2024 Sep 17;42(1):e213854. doi: 10.1136/emermed-2023-213854 (PMC11874362; doi:10.1136/emermed-2023-213854)
Supplement: online supplemental file 2 [file emermed-42-1-s002.pdf]

Supplementary material 2: ED Staff Interview Topic Guide v1.1

N.B. This is a broad topic guide that gives an indication of the type of questions that will be asked within the semi-structured interview. Questions will differ slightly depending on the role of the person being interviewed. Over the course of the data collection period, questions/ ordering of questions may change as we pursue emerging lines of enquiry.

Date:

ID:

Voucher code:

Thank you for agreeing to take part in this interview. We have just run through the consent form, but I would just like to remind you that whatever is discussed today will remain confidential and you won't be identified in any transcripts or subsequent publication of the results. Do you still consent for the interview to be audio-recorded? (Y/N). Do you have any questions before we begin?

- Can you start off by telling me your role, how long you've been in the role and how your role relates to ambulance pre-alerts.

- Thinking back to the last pre-alert call that you dealt with, can you talk me through what happened from when the (red phone) rang (Prompt - tell me about the information you received during the call, understand how they got the information they needed, use of checklists etc.)

- Thinking about the same example, can you tell me what you did in response to the call? (Prompt to understand why they responded like they did, what influenced their decision).

- Can you talk to me about what factors influence how you respond to pre-alerts? (Is this the same for colleagues, has practice changed?)

- Can you think of examples of useful pre-alerts and how these influenced patient care? (Focus on a specific example, what made it effective) / Can you talk to me about the benefits of pre-alerts (ask for examples).

- Can you give me an example of pre-alerts that were not useful and may have had a negative influence on patient care (Focus on specific example). Can you talk to me about the potential risks of pre-alerts (ask for examples).

- Are there any particular kind of conditions/patients who you feel are pre-alerted too often

- Are there any particular kind of conditions/patients who you feel should be pre-alerted more than they are currently?

- Do you think there is variation between paramedics in terms of how pre-alert decisions are made?

- What, if anything, do you think the ED could do to make the pre-alert process easier for ambulance clinicians?

- Do you provide feedback to ambulance staff about their pre-alert decisions?

- What, if anything, do you think would help ambulance clinicians to make better pre-alert decisions?

- (Pick up on anything else that arises during the course of the interview).

- Thank you for talking to us. Is there anything else you'd like to add?

- (Talk about what will happen to the research now, give them chance to ask questions).

Project contact details for further information:

Dr Jaqui Long / Jo Coster , School of Health and Related Research, University of Sheffield, 30 Regent Street, Sheffield S1 4DA Email: prealerts@sheffield.ac.uk / prealerts@sheffield.ac.uk Tel no: 0114 2225441 / 0114 2220854

In the event of a complaint, please contact Dr Fiona Sampson (Principal Investigator), Email: f.c.sampson@sheffield.ac.uk, Tel no: 0114 2220687
