# Supplementary material for: How do emergency departments respond to ambulance pre-alert calls? A qualitative exploration of the management of pre-alerts in UK emergency departments
Source: Emerg Med J. 2024 Sep 17;42(1):e213854. doi: 10.1136/emermed-2023-213854 (PMC11874362; doi:10.1136/emermed-2023-213854)
Supplement: online supplemental file 3 [file emermed-42-1-s003.pdf]

Supplementary table 3: Description of sites and pre-alert processes

Site code, type of site (MTC/TU)	Resus provision.<br><br>Alternative options if not accepted to resus.	Access to resus for ambulance crews	Location of red phone(s). Who answers?	Pre-alert documentation.<br>How information about prealert is communicated to others, including use of documentation	Who is involved in prealert decision-making?<br>Key staff involved in management of alerts.
A – MTC	8 resus beds, with trauma/ high acuity bays nearest ambulance entrance.<br><br>Alternative: initial assessment area	Direct from outside or from usual ED entrance following assessment.<br>Crews only bring direct to resus if this has been agreed on the phone	In resus, but audible throughout the department.<br>Policy is to be answered by consultant, but answered by whoever is nearest.	One form for all calls.<br>Form either goes by patient's bed in resus or is taken to usual ED entrance for the receiving nurse/staff. Other relevant staff informed verbally by call-taker.	Decision made by person answering, with input if needed.<br>Consultant and NIC assigned specifically to resus.
B – TU	5 resus beds, with trauma/high acuity bay nearest ambulance entrance.<br><br>Alternatives: a) 2 high dependency beds; b) initial assessment area	Either direct from outside, or from assessment area.<br>Crews only bring direct to resus if this has been agreed on the phone?	In majors, on main desk where doctors are sitting.<br>Answered by doctor generally, as tend to be nearest person to the phone.	Separate trauma and medical forms.<br>Form taken to resus, high dependency cubicle, or left by ambulance handover bays for nurse receiving ambulance crew. Other relevant staff informed verbally by call-taker.	Decision made by person answering, with input if needed.<br>Consultant has oversight of resus, high dependency and majors,
C – TU	4 resus beds<br><br>Alternatives: a) 4 beds with higher staff/ patient ratio; initial assessment area or assessment on ambulance	Off main corridor into the department only. Crews only bring direct to resus if this has been agreed on the phone. No other access route.	In majors, at NIC desk.<br>Not audible in other areas of department.<br>Answered by NIC when possible, by whoever is nearest if not.	One form for all calls.<br>Form taken to resus or high care area, or given to assessment nurse in usual ED entrance area receiving ambulance crew.<br>Other relevant staff, including HALO, informed verbally by call-taker.	Most decisions made by NIC, with consultant/medical input when needed.<br>Consultant manages resus and high care, others in majors.
D – MTC	5 resus beds, with trauma/high acuity bays nearest ambulance entrance<br><br>Alternatives: a) 6 bed rapid assessment &	Off the main corridor from the usual ED entrance and majors area. Crews only bring to resus if agreed on the phone. No other access route.	2 phones in resus at staff desk; a third phone rings in majors if other two lines engaged.<br>Answered by whoever is nearest who feels	Separate trauma and medical forms in folders.<br>Forms generally remain in folders. Relevant staff, including rapid assessment area staff, receiving nurse & HALO, informed verbally and/or	Decision mostly made by person answering, with additional input if needed. Consultant informed of/approves all decisions re patients NOT accepted to resus.<br>Consultant cover from majors, variably in resus much of time

	treatment area; b) direct to majors		confident to do so, often ODP.	through a 'bleep' system via main switchboard.	
E – MTC	9 resus beds, some of which can be divided, with trauma/high acuity bays nearest ambulance entrance.<br><br>Alternatives: initial assessment area	Immediately off the corridor by the ambulance entrance. Crews can drop in and ask about patients they are concerned about but haven't alerted.<br>Also an entrance from usual ED entrance area/majors	In resus, at NIC desk.<br>Answered by NIC mostly, but whoever is nearest.	One form for all.<br>Forms either goes by patient's bed in resus or may be taken to usual ED entrance area but not consistently – some are left in a pile by the red phone. Other relevant staff in resus informed verbally.	Decision mostly made by person answering, with additional input when needed.<br>Consultant and NIC assigned specifically to resus.<br>Consultant variably involved in decision-making.
F – TU	7 resus beds, with trauma bay nearest ambulance entrance.<br><br>Alternative: initial assessment area	Through usual ED entrance area. Crews can't access without going through pit stop. Crews only bring to resus if agreed on the phone.	In resus, at staff desk.<br>Bell also rings in majors, making them aware of the call.<br>Answered by whoever is nearest, generally NIC or more senior doctor.	One form for all.<br>Forms either go by patient's bed in resus or are taken to usual ED entrance and handed to NIC or doctor. Other relevant staff informed verbally.	Decision mostly made by person answering, with additional input when needed.<br>Consultant cover from majors.

* The term 'usual ED entrance' is used to describe all department's initial assessment and treatment area i.e. where those patients not being taken to resus etc are received.
