# Supplementary material for: How do emergency departments respond to ambulance pre-alert calls? A qualitative exploration of the management of pre-alerts in UK emergency departments
Source: Emerg Med J. 2024 Sep 17;42(1):e213854. doi: 10.1136/emermed-2023-213854 (PMC11874362; doi:10.1136/emermed-2023-213854)
Supplement: online supplemental file 4 [file emermed-42-1-s004.pdf]

Supplementary table 4: Outline description of observation sites and pre-alert processes

Site	Brief description
A-MTC	Department, including resus, often full, and ambulances frequently queuing outside. Some assessment of patients on ambulances at particularly busy times. Hospital Ambulance Liaison Officer (HALO) paramedic also present at these times to help prioritise queues, support ambulance crews.<br>Few computer terminals for resus staff, and complaints about poorly functioning IT. Pre-alert phone rings very loudly, audible across department. Resus has an allocated consultant, nurse in charge and other medical and nursing staff. Information regarding pre-alerts not accepted into resus generally reliably conveyed to staff in main department verbally and with pre-alert form.
B-TU	Department as a whole busy, but resus not usually full, and ambulance crews rarely queueing for long for assessment, even when not pre-alerted. Resus area separate from the rest of the ED, and only staffed when patients present. Pre-alert calls taken and decisions made in main department. Pre-alerts a much smaller part of the overall workload, occurring much less frequently than at other sites during observations. Nurse generally manages patients in resus, with other staffing provided from main department as needed.
C-TU	Department generally full, with ambulances often queueing for long periods. Very overcrowded, with patients frequently assessed and managed in corridor and on ambulances. HALO often on site to facilitate management of queues, sometimes providing additional information on incoming patients. Pre-alert phone inaudible at any distance from nurse in charge's desk a significant problem. Pre-alerts often required significant 'reshuffling' of patients and liaison with multiple staff to make space – nurse in charge has key role in decision-making, with consultant input at times – occasional tensions between logistical and clinical priorities. Information conveyed verbally and pre-alert form taken to receiving area. ED staff have access to ambulance crews' electronic patient records before arrival.
D-MTC	Department generally full, with ambulances often queueing. HALO generally present and with key role in facilitating communication. Rapid assessment area provided an intermediate level of response for some pre-alerts. Three pre-alert phone lines. Core resus staffing included specialist practitioners who had key role in answering phone and treating patients. Variable level of consultant input into resus, depending on individuals and demand. Details of alerts not accepted into resus conveyed verbally, but generally reliably. 'Bleep' system via switchboard used to notify key staff of incoming alerts. Pre-alert paperwork not linked to patient notes. ED staff had access to ambulance crews' electronic patient records before arrival.
E-MTC	Very busy department. Patients generally not held on ambulances but queuing on trolleys with ambulance clinicians along corridors, often for long periods. Large resus area, with capacity for further sub-division of cubicles at busy times. Much smaller initial assessment area for patients not accepted into resus. Crews sometimes came to resus on arrival with non-alerted patients for quick assessment, and this was accepted by resus staff. Resus has allocated consultant, nurse in charge and other medical and nursing staff. Consultant input into decisions varied depending on the individuals and demand. Information regarding pre-alerts not accepted into resus not consistently recorded on forms or conveyed to assessment area staff.
F-TU	Very busy department, with ambulances often queuing, though generally not for long periods. Initial assessment area very busy, with frequent movement through to 'majors' area as beds became free. Pre-alert phone triggers bells in majors area but does not prompt any specific response. Resus largely managed by nurse in charge and 'junior' doctors, including experienced registrars – consultant based in majors provides input depending on resus staff experience level and clinical demand. Some tension observed when pre-alerts not accepted into resus and passed to initial assessment area – concern re risk, ability to manage. Information regarding pre-alerts not accepted into resus conveyed verbally and with pre-alert form.
